# Glyoxalase 1: Emerging biomarker and therapeutic target in cervical cancer progression

**DOI:** 10.1371/journal.pone.0299345

**Published:** 2024-06-13

**Authors:** Ji-Young Kim, Ji-Hye Jung, Soryung Jung, Sanghyuk Lee, Hyang Ah Lee, Yung-Taek Ouh, Seok-Ho Hong

**Affiliations:** 1 Department of Internal Medicine, School of Medicine, Kangwon National University, Chuncheon, Republic of Korea; 2 Department of Life Science, Ewha Womans University, Seoul, Republic of Korea; 3 Department of Obstetrics and Gynecology, School of Medicine, Kangwon National University, Chuncheon, Republic of Korea; 4 Department of Obstetrics and Gynecology, Ansan Hospital, Korea University College of Medicine, Gyeonggi, Republic of Korea; 5 KW-Bio Co., Ltd, Chuncheon, Republic of Korea; Martin Luther University, GERMANY

## Abstract

**Introduction:**

Cervical cancer presents a significant global health challenge, disproportionately impacting underserved populations with limited access to healthcare. Early detection and effective management are vital in addressing this public health concern. This study focuses on Glyoxalase-1 (GLO1), an enzyme crucial for methylglyoxal detoxification, in the context of cervical cancer.

**Methods:**

We assessed GLO1 expression in cervical cancer patient samples using immunohistochemistry. *In vitro* experiments using HeLa cells were conducted to evaluate the impact of GLO1 inhibition on cell viability and migration. Single-cell RNA sequencing (scRNA-seq) and gene set variation analysis were utilized to investigate the role of GLO1 in the metabolism of cervical cancer. Additionally, public microarray data were analyzed to determine GLO1 expression across various stages of cervical cancer.

**Results:**

Our analysis included 58 cervical cancer patients, and showed that GLO1 is significantly upregulated in cervical cancer tissues compared to normal cervical tissues, independent of pathological findings and disease stage. *In vitro* experiments indicated that GLO1 inhibition by S-p-bromobenzylglutathione cyclopentyl diester decreased cell viability and migration in cervical cancer cell lines. Analyses of scRNA-seq data and public gene expression datasets corroborated the overexpression of GLO1 and its involvement in cancer metabolism, particularly glycolysis. An examination of expression data from precancerous lesions revealed a progressive increase in GLO1 expression from normal tissue to invasive cervical cancer.

**Conclusions:**

This study highlights the critical role of GLO1 in the progression of cervical cancer, presenting it as a potential biomarker and therapeutic target. These findings contribute valuable insights towards personalized treatment approaches and augment the ongoing efforts to combat cervical cancer. Further research is necessary to comprehensively explore GLO1’s potential in clinical applications.

## Introduction

Cervical cancer, a prevalent and overwhelming malignancy among women, presents a significant global health challenge. Although early detection methods such as HPV (Human papillomavirus) testing and PAP tests can identify precancerous lesions on the cervix, the disease remains highly prevalent due to its unclear pathogenesis. HPV infection, especially high-risk HPV types, is a recognized risk factor for developing cervical cancer [1]. The most common genotypes being human papillomavirus types 16 and 18 (HPV16/18) [2], and HPV infection alters the metabolism of tumor cells, causing infected cells to favor glycolysis for energy production, even in oxygen-rich environments. This shift not only supports the rapid proliferation of cancer cells but also contributes to an acidic tumor microenvironment, potentially enhancing cancer cell invasiveness [3]. These metabolic adaptations are key to the progression of cervical cancer, from the early stages of HPV infection to the development of precancerous lesions and ultimately invasive cancer. A deeper understanding of these pathways is essential to develop early diagnosis and targeted treatment strategies to reduce the global burden of cervical cancer.

The glyoxalase system, consisting of glyoxalase 1 (GLO1) and glyoxalase 2 (GLO2), catalyzes the conversion of glycolytic methylglyoxal (MG) to non-toxic S-D lactate, of which GLO1 represents a tumor survival strategy by inhibiting the accumulation of cytotoxic MG in highly metabolized tumor tissues [4]. Overexpression of GLO1 has been reported in various tumor tissues, including lung, stomach, and brain, and cancer patients with high expression of GLO1 have been shown to have significantly lower survival compared to patients with low expression [5–8]. Furthermore, overexpression of GLO1 in clinical chemotherapy has been shown to reduce the cytotoxicity of anticancer drugs, contributing to multi-drug resistance, which plays an important role in cancer progression and prognosis [9–11]. In terms of cancer stem cells (CSCs), it has been reported that aldehyde dehydrogenase 1-positive breast CSCs with high activity of GLO1 exhibit high aggressiveness, indicating that GLO1 is essential for the survival of CSCs [12–14]. All these findings suggest that GLO1 could be a potential target for tumor therapy and anticancer drug development. Most of the research on GLO1 in gynecologic cancers has been conducted in breast cancer, and the role of GLO1 in cervical cancer has not been explored. Cervical cancer has a clear cause and the focus is on prevention rather than therapeutic intervention [15]. It still remains the most common and overwhelming malignancy in women, highlighting the need for identifying new biomarkers and improving treatment options to effectively manage cervical cancer clinically.

Numerous GLO1 inhibitors have been developed to induce an increase in MG levels in cancer cells and can be applied as anti-tumor agents [16]. S-p-bromobenzylglutathione cyclopentyl diester (BBGC), a highly cell permeable GLO1 inhibitor, was first proposed by Vince and Ward in 1969 [16, 17]. It was reported that cell death by BBGC was observed in human lung cancer NCI-H522 and DMS114 cells with high expression of GLO1, while it was not observed in A549 cells with low expression, suggesting that the sensitivity of BBGC is positively correlated with the activity of GLO1 [18]. Furthermore, in a study on drug-resistant cancer, it was reported that in human hepatocellular carcinoma with high expression of GLO1, combined treatment with GLO1 inhibitor can improve the sensitivity of cancer cells to drugs compared to GLO1 inhibitor alone [19]. These data demonstrate the potential of the GLO1 system as a diagnostic and therapeutic target and suggest that strategies to modulate GLO1 may increase therapeutic efficacy.

In this study, we aimed to investigate whether the expression of GLO1 is involved in the progression of cervical cancer. Immunohistochemistry showed the elevated expression of GLO1 in cervical cancer tissues compared with cervix without tumor from the different participants. Downregulation of GLO1 with small interfering RNA (siRNA) in human cervical cancer cell line (HeLa) resulted in a significant reduction in cell viability and migration. Furthermore, clinical analysis of patient samples revealed that the expression of GLO1 in cervical tumor tissues varies depending on the stage of diagnosis. Our findings suggest that high expression of GLO1 in cervical cancer is closely associated with the progression of tumor cells, and propose it as a promising therapeutic target as well as a potential biomarker for detection and treatment of cervical cancer.

## Materials and methods

### 1. Immunohistochemistry

Cervical cancer patient samples/specimens were obtained with approval from the Institutional Review Board (IRB) of Kangwon National University Hospital (KNUH-A-2022-07-010). The biospecimens and data used for this study were provided by the Biobank of Kangwon University Hospital, a member of the Korea Biobank Network. Tissues stored in the Biobank, along with anonymized participant information, are all preserved with prior consent from the patients. The collection dates for the participant tissues span from January 1, 2021, to December 31, 2022. Following this phase, for the purpose of our research, we accessed cervical, uterine, and ovarian cancer patient samples from January 1, 2023, to March 31, 2023. Additionally, for comparison with non-cancerous tissue, patients who underwent surgery for uterine fibroids and adenomyosis during the same period were enrolled to obtain tissues. Information regarding individual medical records of participants is inaccessible, and only anonymized patient information can be obtained from the Biobank. All participants providing specimens for storage in the Biobank have given prior consent, which has been documented in written form and is held by the Biobank.

Paraffin-embedded tissue sections were deparaffinized and rehydrated. The slides underwent antigen retrieval using a buffer (0.01 M sodium citrate, pH 6) and were quenched in 3% H_2_O_2_ for 15 min. For GLO1 staining, sections were incubated with GLO1 antibody (ab171121) overnight at 4°C. The labeled antigen was visualized using DAKO secondary antibody (DAKO) and DAB solution (ab64238; Abcam Inc, Toronto, ON, Canada). Sections were counterstained with hematoxylin and then mounted. The intensity of GLO1 protein staining in the tissues was evaluated on a scale of 0 to 5, with 0 indicating no staining (negative), 1 for weak staining, 2 to 3 for moderate staining, and 4 to 5 for strong staining. Low expression was defined as negative or weak staining, and high expression as moderate or strong staining. Each section was independently evaluated by three researchers.

### 2. Cell lines

Human cervical cancer cell line (HeLa-CCL2) was purchased from ATCC (Manassas, VA, USA). Cells were cultured in DMEM/High glucose (Thermo Fisher Scientific, Inc., Waltham, MA, USA), supplemented with 10% fetal bovine serum (FBS) (Thermo Fisher Scientific, Inc., Waltham, MA, USA) and 1% penicillin-streptomycin (Sigma-Aldrich, St. Louis, MO, USA). The cultures were maintained in a humidified atmosphere at 37°C with 5% CO_2_. Upon reaching 80–90% confluency, the cells were seeded in 96-well plates for the MTT assay.

### 3. MTT assay

24 hours post seeding of the HeLa cells into 96-well plates, cells were treated with GLO1 inhibitor (SML1306; Sigma-Aldrich, St. Louis, MO, USA) at concentrations of 0 (control), 8 μM, and 10 μM for 48 h. After treatment, the cultures were replaced with serum-free medium containing MTT reagent (ab211091; Abcam Inc, Toronto, ON, Canada) and incubated for 3 h. The cells were subsequently treated with MTT solvent for 15 min at room temperature. Absorbance at 570 nm was measured using a microplate reader.

### 4. Wound healing assay

HeLa cells were seeded into 6-well plate culture dishes (2x10^5^ cells/well). The cells were treated with 0 (control), 8 μM, and 10 μM of GLO1 inhibitor and GLO1 siRNA treated for 24 h. For the wound healing assay, lines were created by scraping the cell monolayer with a 200 μl pipette tip. Photos were taken under a microscope at 24, 48, and 72 h to document the closure of the wound.

### 5. siRNA transfection

*In vitro* siRNA transfection was performed using lipofectamine 3000 reagent (Thermo Fisher Scientific) and GLO1 siRNA oligonucleotide at a final concentration of 20 nM base on the manufacturer’s instructions. Cells were collected 48 and 72 hours after transfection and RNA and protein were isolated for experiments. The following siRNAs were used: NC siRNA, *AccuTarget*^*TM*^ Negative control siRNA (Bioneer, Daejeon, Korea), GLO1 siRNA A (GAC UCU AGU GGA AGA CCU; Bioneer), and GLO1 siRNA B (AAG GUC UUC CAC UAG AGU C; Bioneer).

### 6. RNA extraction and quantitative real time PCR

Total RNA was extracted from HeLa cells using a RNeasy Mini kit (Qiagen, Duesseldorf, Germany) and cDNA was synthesized using TOPscrip^TM^ RT DryMIX (Enzynomics, Daejeon, Korea). PCR amplification was performed using a Step One Plus real time PCR system (Applied Biosystems, Warrington, UK) with TOPreal^TM^ qPCR 2X PreMIX (Enzynomics). The mRNA expression was normalized to an internal control GAPDH. The expression levels of the target gene mRNAs were calculated by comparing them to the expression levels of GAPDH using the 2^-ΔΔCt^ method. The primer sequences are listed in Table 1.

**Table 1:** Primer sequences for real-time RT-PCR.

Gene		Sequence (5’-3’)	Size (bp)
*BCL2*	F	TCGCCCTGTGGATGACTGA	134
	R	CAGAGACAGCCAGGAGAAATCA	
*BAX*	F	TGGCAGCTGACATGTTTTCTGAC	195
	R	TCACCCAACCACCCTGGTCTT	
*GLO1*	F	ATGCGACCCAGAGTTACCAC	132
	R	CCAGGCCTTTCATTTTACCA	
*GAPDH*	F	GGCATGGACTGTGGTCATGA	87
	R	TGCACCACCAACTGCTTAGC	

F; Forward, R; Reverse

### 7. Single-cell RNA sequencing (scRNA-seq) data analysis

The scRNA-seq data from tumor and adjacent normal samples of cervical squamous cell carcinoma patients were downloaded from ArrayExpress (accession number E-MTAB-11948) [20]. We processed the scRNA-seq data using Seurat (v4.1.1) [21], focusing on gene-count matrices. For quality control, cells with a gene count ≤ 200 or a percentage of mitochondrial genes ≥ 20% were excluded as low-quality cells. The QC-passed data were log-normalized using the "NormalizeData" function. Next, we scaled the data by regressing out the percentage of mitochondrial genes and cell cycle scores with the "ScaleData" function. The cell cycle scores (S and G2M scores) were calculated using the "CellCycleScoring" function, based on the expression levels of S/G2M phase markers from Seurat. Principal component analysis (PCA) was performed on the scaled data using the top 2000 variable genes. For batch correction and sample integration, we utilized Harmony (v0.1.1) [22]. Unsupervised clustering was executed using "FindNeighbors" and "FindClusters" functions, with the results visualized via the "RunUMAP" function. Prior to further analysis, Scrublet (v0.2.3) [23] was employed to identify and remove a doublet cluster (**S1A and S2B Figs**). Cell types for each cluster were annotated based on canonical marker genes and the top-ranked differentially expressed genes (**S1C Fig**). Pathway activity score for each cell was calculated from the scRNA-seq data. We used GSVA (v1.42.0) [24] to calculate the gene set enrichment score, often regarded as the pathway activity, for each cell and for each pathway. Wikipathways (v2023.1) [25] downloaded from MSigDB [26, 27] was used as a pathway collection. Then, GSVA scores for normal and tumor epithelial cells were compared using limma (v3.50.3) [28] to identify gene sets differentially regulated between the two conditions. Pathways with an adjusted *P-value* (Benjamini-Hochberg procedure) < 0.01 were considered as significant. The Wilcoxon Rank Sum test was used to examine differential expression between normal and tumor epithelial cells.

### 8. Gene Set Variation Analysis (GSVA)

GSVA (v1.42.0) [24] was employed to calculate the gene set enrichment scores for ach cell. We downloaded Wikipathways gene sets (v2023.1) [25] from MSigDB [26, 27] to serve as the reference gene sets. The GSVA scores were then utilized in limma (v3.50.3) [28] to identify gene sets differentially regulated between normal and tumor epithelial cells. Pathways with an adjusted *p-value* < 0.01 were considered significant.

### 9. Microarray data analysis

The microarray data for normal, cervical intraepithelial neoplasia (CIN1 to CIN3), and cervical cancer tissue samples were downloaded from the Gene Expression Omnibus (accession number GSE63514) [29]. Raw expression values were normalized using the Robust Multi-array Average (RMA) method with the affy package in R (version 1.72.0) [30]. Differential expression across 5 cancer subtypes was examined by the Kruskal-Wallis test. We also performed pairwise comparisons using the Dunn’s test (dunn.test v1.3.5) with the adjusted p-value according to the Benjamini-Hochberg correction.

### 10. Statistical analysis

Values representing the expression of GLO1 in cervical cancer are expressed as means ± standard error of the mean (SEM). Student’s *t*-test was used for comparisons between two groups, and one-way ANOVA was used for comparisons between two or more groups. Statistical analysis was performed using GraphPad Prism v.9 software (GraphPad Software Inc, San Diego, CA, USA), and a *P*-value of less than 0.05 was considered statistically significant.

## Results

### Populations

A total of 73 gynecological cancer patients were enrolled, comprising 58 cases of cervical cancer, 7cases of uterine cancer, and 8 cases of ovarian cancer. Normal tissue (cervix, endometrium, and ovary) was obtained and analyzed from 16 patients with benign uterine disease who underwent hysterectomy with both salpingo oophoretomy and were pathologically confirmed to have non-cancerous lesions (Table 2). Analysis was also conducted on specimens from 7 patients with uterine cancer and 8 patients with ovarian cancer. The average age of the patients was 57.4±11.8 years for uterine cancer and 57.76±12.85 years for ovarian cancer. The histological type of the uterine cancer was entirely endometrioid carcinoma, while the ovarian cancer included various pathologic types, including 4 cases of high-grade serous cell carcinoma. Immunohistochemistry specifically for glyoxalase 1 was conducted on specimens from all participating patients.

**Table 2:** Clinical characteristics of patients with cervical, uterine and ovarian cancer.

Variables	Cervical Cancer (n = 58)	Uterine Cancer (n = 7)	Ovary Cancer (n = 8)	Normal (n = 16)
Age (years)	55.1±15.1	57.4±11.8	57.76±12.85	49.7±8.0
Stage				
I	18 (31.03%)	6 (85.71%)	4 (50.00%)	
II	16 (27.59%)			
III	8 (13.79%)	1 (14.29%)	3 (37.50%)	
IV	16 (27.59%)		1 (12.50%)	
Histology				
Squamous Cell Carcinoma	52 (89.66%)			
Adenocarcinoma	6 (10.34%)			
Endometrioid carcinoma		7 (100.0%)		
High-grade serous cell carcinoma			4 (50.00%)	
Mucinous carcinoma			2 (25.00%)	
Endometrioid carcinoma			1 (12.50%)	
Clear cell carcinoma			1 (12.50%)	

Note: Data available for 73 patients with cervical, uterine and ovarian cancer. SD, standard deviation.

### GLO1 is upregulated in cervical cancer tissues compared to normal cervical tissues

**Fig 1:**
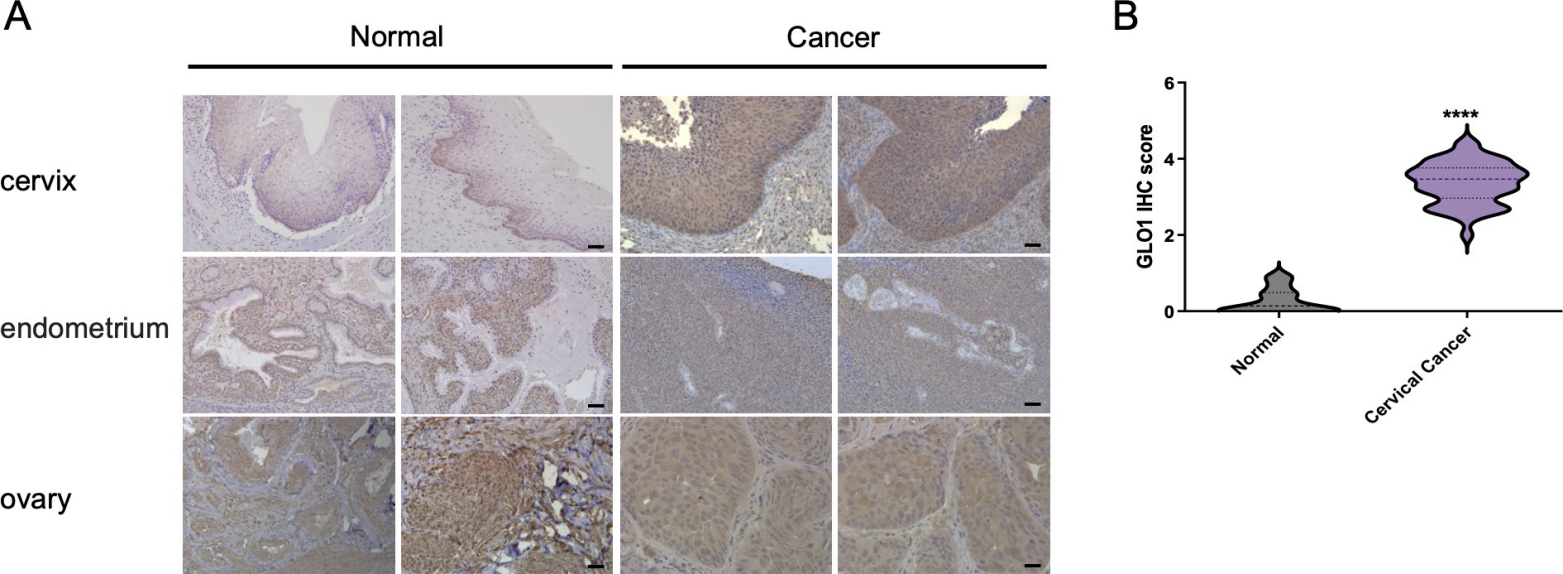
Analysis of GLO1 expression in gynecological cancer tissue through IHC staining. (A) Representative GLO1 staining of cervix, endometrium and ovary tissues (scale bar, 50 μm). (B) GLO1 IHC score comparison between cervical cancer and normal tissues. Statistical analysis of data was performed using the *t*-test (**** *P*<0.0001).

We first sought to investigate the expression analysis of GLO1 in gynecologic cancer patient tissues. GLO1 expression was upregulated in cervical cancer tissues compared with cervix without tumor from the different participants and was mainly localized in the cytoplasm and cell membrane. As in cervical cancer tissue, GLO1 upregulation was not observed in endometrial and ovarian cancers, and no significant changes in GLO1 were identified between normal and cancerous tissues **(Fig 1A)**. Furthermore, the comparison of staining intensity according to IHC scores showed that 3.372±0.340 was measured in cervical cancer tissues, which was higher than the normal 0.260±0.164 **(Fig 1B)**, indicating that the expression of GLO1 was significantly upregulated in cervical cancer tissues.

### Inhibiting GLO1 affects the proliferation and apoptosis of cervical cancer cells

**Fig 2:**
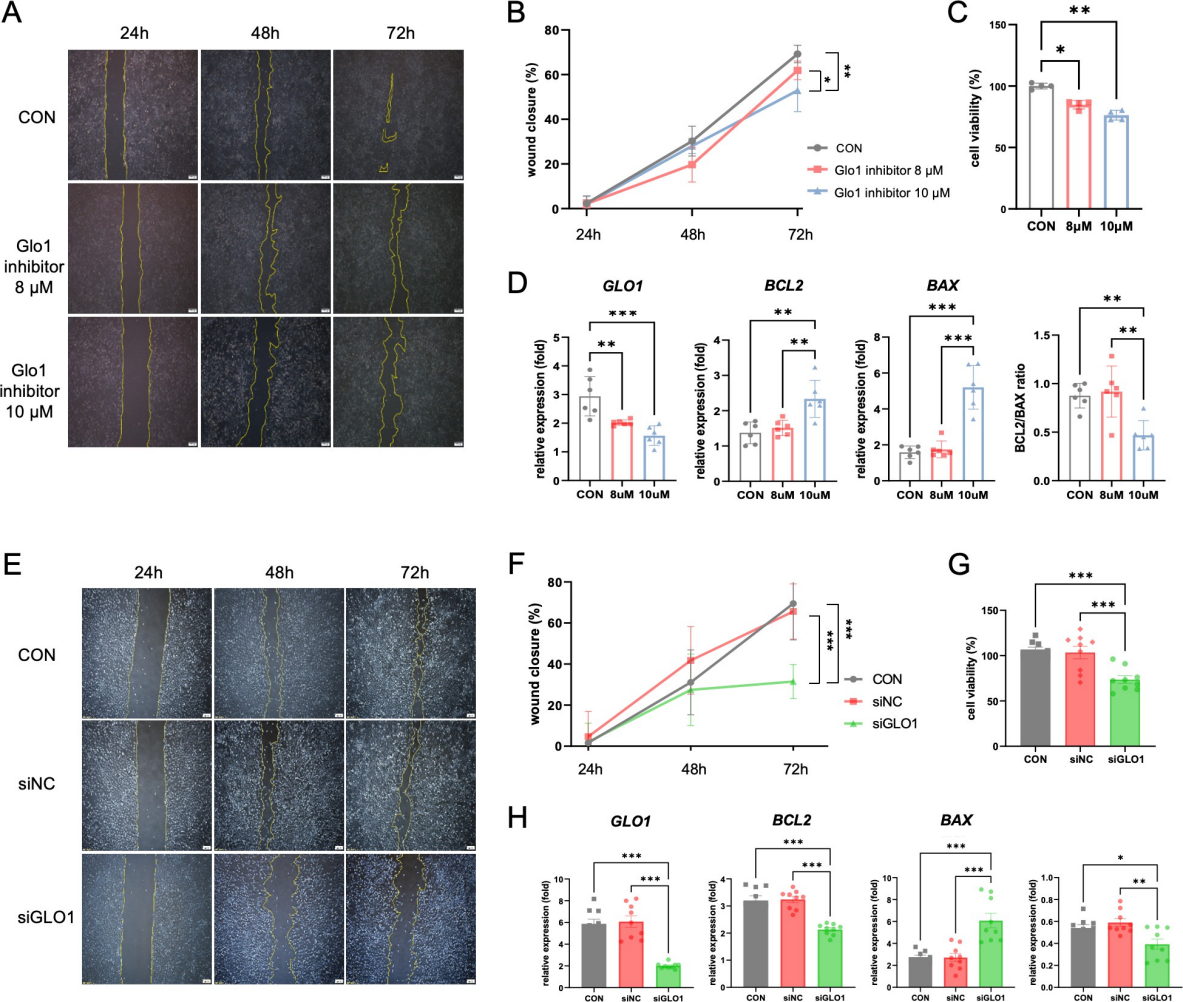
Effect of GLO1 inhibition and knockdown on viability and migration of HeLa cells. (A) The wound healing assay evaluating the effect of GLO1 inhibitor (BBGC) on the HeLa cell migration ability at 24h, 48h and 72 hours (scale bar, 200 μm). (B) Quantitative analysis of the wound closure of HeLa cells. (C) The effect of GLO1 inhibitor (8 μM and 10 μM) on HeLa cell viability by MTT assay. (D) After 48h of treatment with GLO1 inhibitor, the mRNA levels of *GLO1*, *BCL2* and *BAX* in the HeLa cell lysates were analyzed by qPCR. (E) The wound healing assay evaluating the effect of GLO1 knockdown with siRNA on HeLa cell migration ability at 24, 48, and 72 h (scale bar, 200 μm). (F) Quantitative analysis of the wound closure of HeLa cells. (G) The effect of GLO1 knockdown on HeLa cell viability by MTT assay. (H) The mRNA levels of *GLO1*, *BCL2* and *BAX* in HeLa cell lysates after 48 h of GLO1 knockdown using GLO1 siRNA were analyzed by qPCR. Data are presented as mean ± SD. *P<0.05, **P<0.01. Statistical analysis of data was performed using the one-way ANOVA (***P<0.001; **P<0.01; *P<0.05) (three independent experiments).

Next, we investigated cancer cell death and proliferation upon inhibition of GLO1 in cervical cancer cells to determine whether upregulation of GLO1 is involved in tumor progression. In Hela cells treated with 8 μM and 10 μM of Glo1 inhibitor (BBGC), we observed the wound closure rate for 72 hours using the wound healing assay, and found that the Glo1 inhibitor reduced the proliferation of cervical cancer cell in a dose- and time-dependent manner **(Fig 2A and 2B)**. We also measured cell viability by MTT assay to evaluate the effect of GLO1 inhibition on the viability of HeLa cells. We found that high concentration treatment of Glo1 inhibitor induced the expression of apoptosis-related genes and further decreased the viability of the cells **(Fig 2C and 2D**). We further observed a decrease in proliferation and viability of cervical cancer cells by silencing GLO1 mRNA using siRNA **(Fig 2E–2G)**, which might be positively correlated with an increased expression of apoptosis-related genes **(Fig 2H)**.

### scRNA-seq analysis of adjacent normal and tumor samples from cervical squamous cell carcinoma patients (E-MTAB-11948)

**Fig 3:**
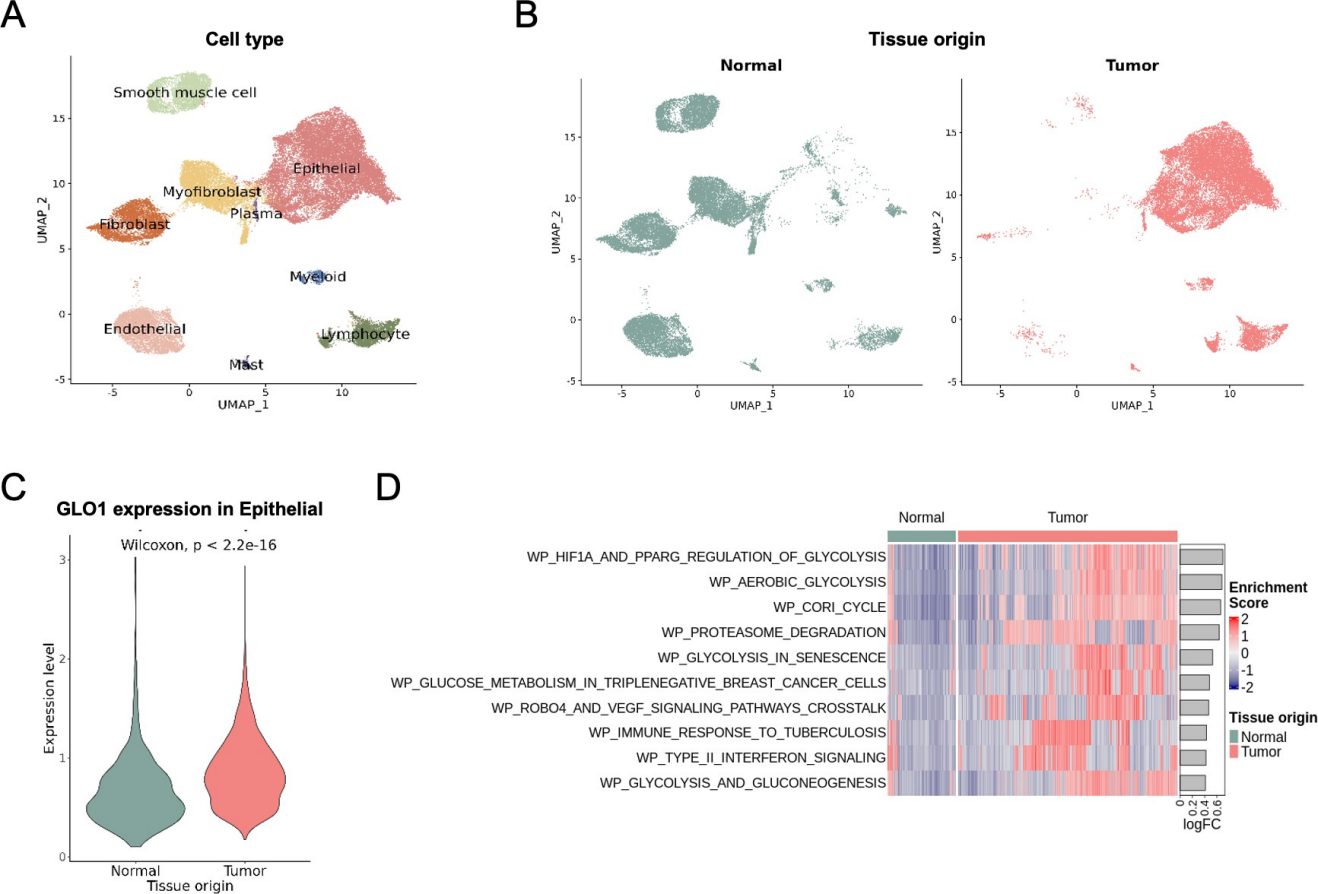
Single-cell analysis of the expression of Glo1 in adjacent normal and tumor samples from patients with cervical squamous cell carcinoma. (A) UMAP plot of the 44,848 cells, colored by cell types. (B) Distribution of 23,636 normal and 21,212 tumor cells in the UMAP plot. (C) GLO1 expression in epithelial cells from normal and tumor tissues. Cells not expressing GLO1 were excluded, resulting in 512 normal and 9,883 tumor cells. (D) Heatmap of GSVA enrichment scores for Wikipathways gene sets. Each column represents epithelial cells from normal and tumor tissues. Top 10 pathways are shown according to the logFC of average GSVA scores between tumor and normal epithelial cells.

We sought to provide additional information to expand *in vitro* findings by examining publicly available gene expression data for cervical cancer at both the single cell and bulk levels. We discovered scRNA-seq data for tumor-normal paired tissues from two patients (ArrayExpress E-MTAB-11948). After performing quality control, normalization, and integration, we successfully identified epithelial, stromal, and various immune cell types (**Fig 3A**). Epithelial cells were notably predominant in the tumor tissue (**Fig 3B**). *GLO1* expression was found to be significantly higher in tumor epithelial cells compared to normal epithelial cells (**Fig 3C**). We then analyzed which pathways were differentially regulated between normal and tumor epithelial cells. Cell-specific pathway activities were determined using the GSVA algorithm for Wikipathways, revealing that many pathways enriched in tumor cells were associated with the glycolysis process (**Fig 3D**). Overall, the single-cell data confirmed that *GLO1* is overexpressed in cervical tumor samples, potentially playing a crucial role in cancer metabolism through the glycolysis pathway.

### GLO1 expression is positively correlated with cervical tumor progression

**Fig 4:**
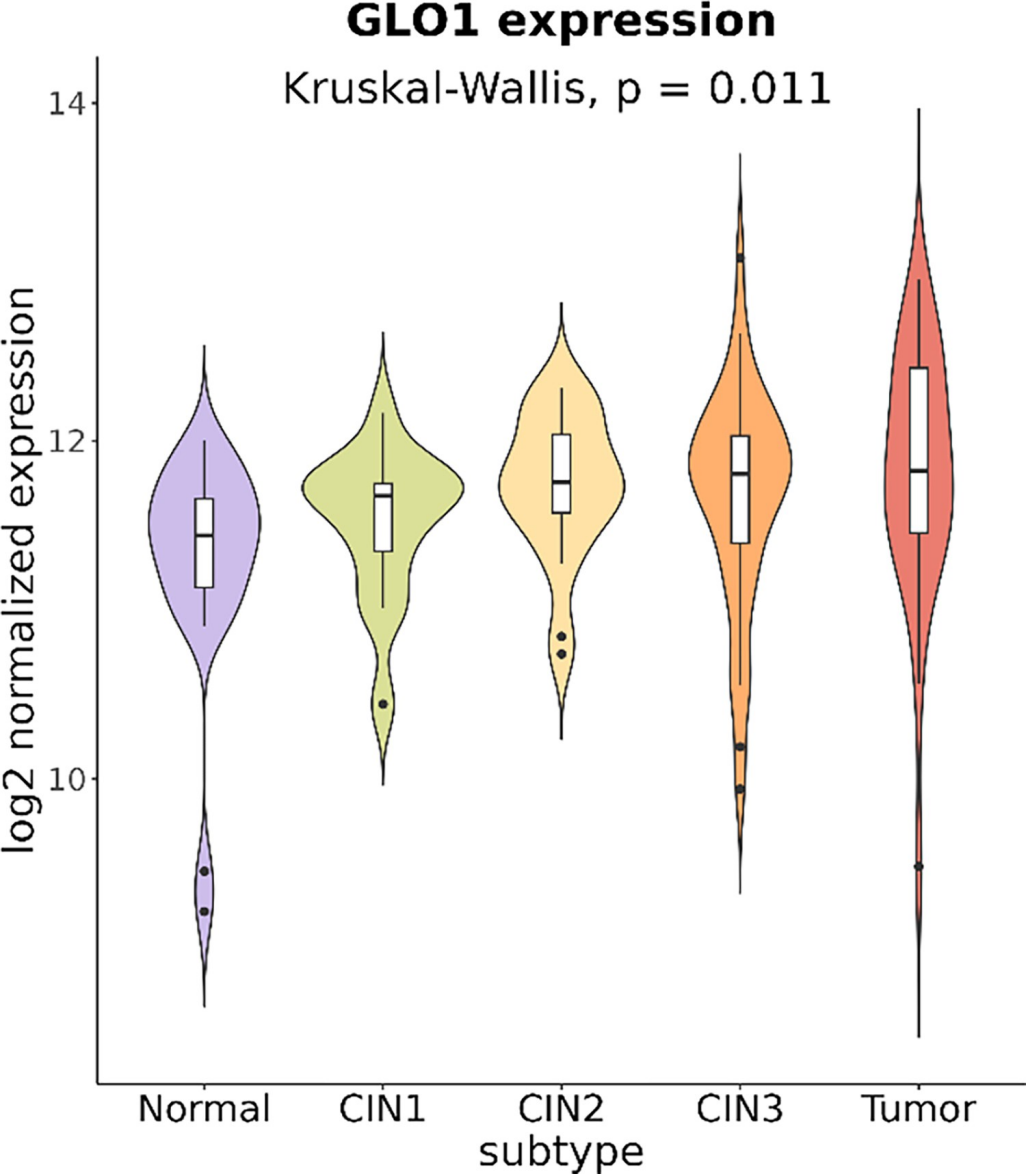
GLO1 expression in normal, cervical intraepithelial neoplasia (CIN1 to CIN3), and cervical cancer tissue samples by microarray analysis. Microarray data from the GEO database (GSE63514) were downloaded and visualized in the violin plots for 24 normal, 14 CIN1, 22 CIN2, 40 CIN3, and 28 cervical cancer samples. The Kruskal-Wallis test was used for global comparison across 5 subtypes, where the Dunn’s test was used for pairwise comparisons.

Next, we investigated the association of GLO1 expression with cancer progression by analyzing public expression data from cervical cancer samples across different tumor stages. The GEO GSE63514 microarray data set included 24 normal, 14 cervical intraepithelial neoplasia (CIN) 1, 22 CIN2, 40 CIN3, and 28 cervical cancer samples. We observed that *GLO1* expression progressively increased from the normal to CIN1, CIN2, CIN3, and tumor sample groups with statistical significance (p = 0.011, Kruskal-Wallis test) (**Fig 4**). Pairwise comparisons using the Dunn’s test showed that *GLO1* expression of the normal group is significantly different from those of the CIN2, CIN3, and tumor subtypes. Our analysis independently confirms that *GLO1* is overexpressed in cervical tumors, likely in a progression-dependent manner.

## Discussion

In this study, we analyzed tissue specimens from 58 cervical cancer patients and found that GLO1 was significantly upregulated in cervical cancer tissue compared to normal, irrespective of the pathological findings and stage. The findings are consistent with proteomic profiling data from the Human Protein Atlas (v23.proteinatlas.org), which ranks GLO1 as one of the 10 most important proteins on a scale of 0–100 in cancer prediction models (**S2A Fig**). In the analysis of a pan-cancer protein panel for cervical cancer prediction, GLO1 was found to be the most important among seven key proteins (**S2B Fig**).

Of note, microarray results obtained from the GEO database showed a positive correlation between the expression of GLO1 and the stage of CIN in epithelial intraepithelial dysplasia, a precancerous stage of cervical cancer. In a previous study, upregulation of GLO1 was identified in high-grade prostate intraepithelial neoplasia (HGPIN), a precursor to invasive cancer, and has been proposed as a novel diagnostic marker to identify precancerous lesions [31]. Reprogramming of energy metabolism has been reported as a hallmark of tumor progression, and it is therefore interpreted that metabolic adaptations associated with the expression of GLO1 may play an essential role in the early stages of tumorigenesis [16, 32]. Although our study is limited by the relatively small sample size, which prevented us from identifying significant differences in the expression of GLO1 between different stages of cervical cancer (early versus advanced or metastatic), we demonstrated that GLO1 may be a target for chemotherapeutic intervention in the early stage of disease in cervical cancer through public gene expression data.

We demonstrated the anti-proliferative and anti-metastatic effects of GLO1 inhibition on HeLa cells and suggested that modulation of GLO1 could be a target for chemotherapy of cervical cancer. Despite the limitation of requiring high expression and activity of GLO1, GLO1 inhibitors have been useful in evaluating treatment outcomes in many tumors, including cervical cancer [9, 33, 34]. Strategies to inhibit GLO1 via the cytotoxicity of MG require an understanding of the effect of MG concentration on cellular function. Low mM concentrations of MG have been reported to have anti-cancer activity, either by blocking cell cycle progression or contributing to cell death through the regulation of apoptosis-related genes [34–36]. On the other hand, heat shock proteins modified by accumulated MG are involved in cell proliferation, invasion, and metastasis, and high levels of MG in GLO1-depleted breast cancer cells increased the likelihood of tumor formation and metastasis, indicating a positive correlation between MG and cancer cell aggressiveness [37–39]. These seemingly contradictory results suggest that determining the concentration of MG is important for inhibitory strategies of GLO1. Based on the therapeutic benefits of inhibiting GLO1 in cervical cancer demonstrated in this study, further investigation of cancer cell growth and survival in response to MG may provide opportunities for major therapeutic breakthroughs in areas of clinical unmet need.

There were several limitations to our study. We did not explore GLO1’s potential as a biomarker in other biological samples, such as patient serum or urine, which warrants further investigation. Despite these limitations, our study possesses several strengths. Primarily, it contributes to the identification of a novel biomarker, GLO1, in cervical cancer. Our research highlights the significant upregulation of GLO1 in cervical cancer tissues, supporting its potential as a valuable biomarker for tumor progression and as a target for cancer therapy. Moreover, we employed a comprehensive methodology that integrates clinical analysis of patient samples, *in vitro* experiments with cell lines, single-cell RNA sequencing (scRNA-seq) data analysis, and examination of public gene expression data. This multifaceted approach offers a thorough understanding of GLO1’s role in cervical cancer, thereby strengthening the credibility of our findings.

## Conclusion

Overall, our comprehensive analysis underscores the pivotal role of GLO1 in the progression of cervical cancer, providing fresh perspectives on its viability as both a therapeutic target and biomarker for this life-threatening disease. Grasping the molecular intricacies of cervical cancer is essential for devising novel diagnostic and treatment approaches. Our findings make a significant contribution to the current endeavors aimed at combating cervical cancer.

## Supporting information

S1 Fig Single cell clusters and marker expression. (A and B) UMAP plot of all (45,048) cells, colored by (A) clusters or (B) Scrublet result. Cluster 17 (dashed circle) was regarded as a doublet cluster, which was excluded from further analyses. (C) Average gene expression of selected marker genes as follows: Epithelial (CDKN2A, EPCAM, CD24, CDH1), Endothelial (PECAM1, CDH5, ENG), Fibroblast (COL1A2, COL3A1, DCN), Myofibroblast (Simultaneous expression of fibroblast and smooth muscle cell markers), Smooth muscle cell (ACTA2, ACTG2, TAGLN), Lymphocyte (CD2, CD3D, CD3E, NKG7), Myeloid (CD163, CD68, LYZ, CSF3R), Mast (MS4A2, CPA3, TPSAB1), and Plasma (IGHG1, JCHAIN).
(DOCX)

S2 Fig Expression analysis of GLO1 protein in a predictive model of human cervical cancer. (A) Volcano plot of average expression differences of proteins specifically expressed in cervical cancer and Lollipop plot of protein rankings from 0–100 in importance in a cancer prediction model. (B) Pan-cancer protein panel analysis of 7 selected proteins for cervical cancer prediction.
(DOCX)
