# Supplementary material for: Glyoxalase 1: Emerging biomarker and therapeutic target in cervical cancer progression
Source: PLoS One. 2024 Jun 13;19(6):e0299345. doi: 10.1371/journal.pone.0299345 (PMC11175447; doi:10.1371/journal.pone.0299345)
Supplement: S1 Fig — (A and B) UMAP plot of all (45,048) cells, colored by (A) clusters or (B) Scrublet result. Cluster 17 (dashed circle) was regarded as a doublet cluster, which was excluded from further analyses. (C) Average gene expression of selected marker genes as follows: Epithelial (CDKN2A, EPCAM, CD24, CDH1), Endothelial (PECAM1, CDH5, ENG), Fibroblast (COL1A2, COL3A1, DCN), Myofibroblast (Simultaneous expression of fibroblast and smooth muscle cell markers), Smooth muscle cell (ACTA2, ACTG2, TAGLN), Lymphocyte (CD2, CD3D, CD3E, NKG7), Myeloid (CD163, CD68, LYZ, CSF3R), Mast (MS4A2, CPA3, TPSAB1), and Plasma (IGHG1, JCHAIN). (DOCX) [file pone.0299345.s001.docx]

**Supporting Information for**

**Glyoxalase 1: Emerging Biomarker and Therapeutic Target in Cervical Cancer Progression**

Ji-Young Kim^1^, Ji-Hye Jung^1^, Soryung Jung^2^, Sanghyuk Lee^2^, Hyang Ah Lee^3^, Yung-Taek Ouh^3,4^*, Seok-Ho Hong^1,5^*

^1^ *Department of Internal Medicine, School of Medicine, Kangwon National University, Chuncheon, Republic of Korea*

^2^ *Department of Life Science, Ewha Womans University, Seoul, 03760, Republic of Korea*

^3^ *Department of Obstetrics and Gynecology, School of Medicine, Kangwon National University, Republic of Korea*

^4^ *Department of Obstetrics and Gynecology, Ansan Hospital, Korea University College of Medicine, Gyeonggi, Republic of Korea*

^5^ *KW-Bio Co., Ltd, Chuncheon, Republic of Korea*

**
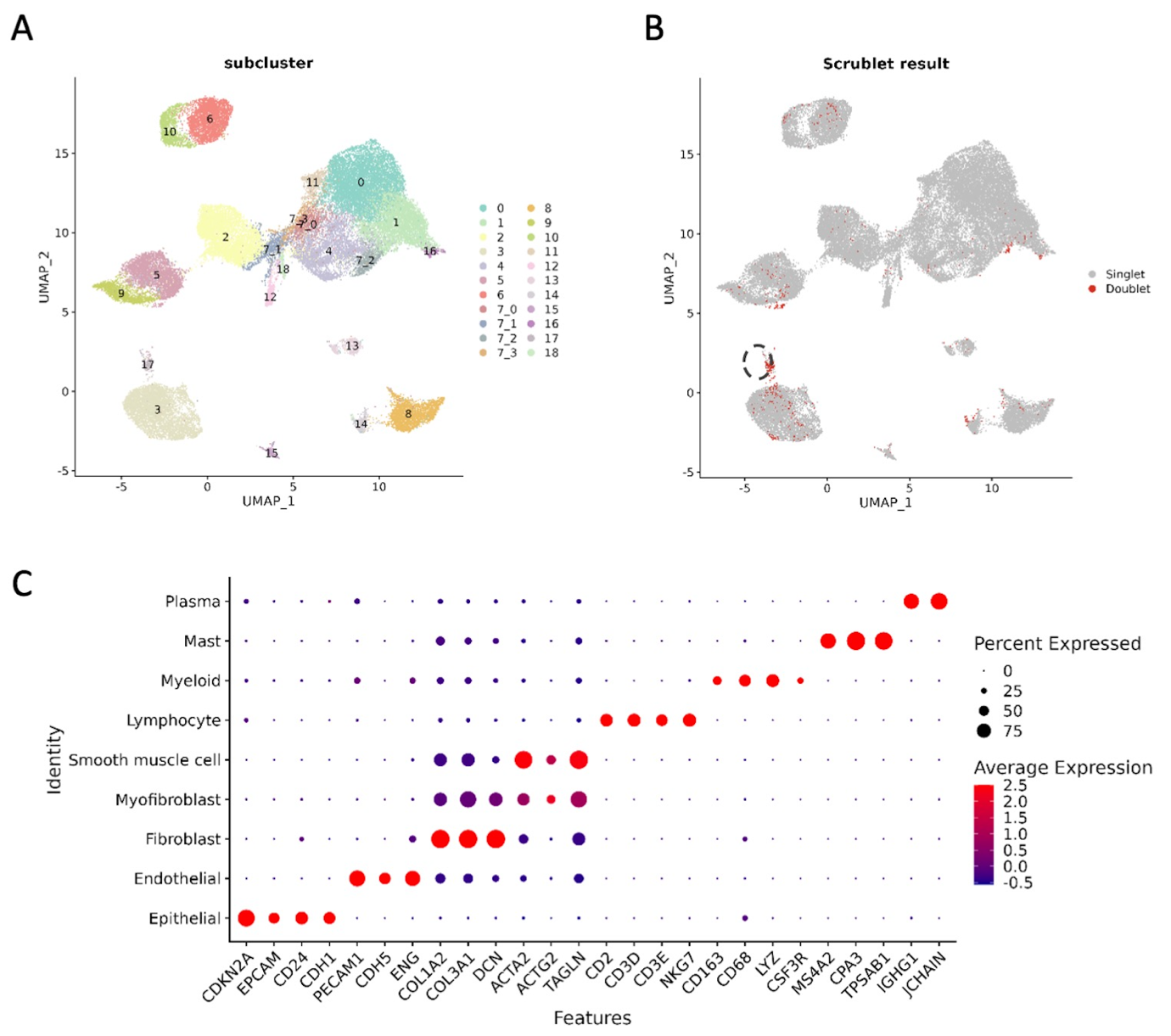
**

**Fig S1. Single cell clusters and marker expression.** (A and B) UMAP plot of all (45,048) cells, colored by (A) clusters or (B) Scrublet result. Cluster 17 (dashed circle) was regarded as a doublet cluster, which was excluded from further analyses. (C) Average gene expression of selected marker genes as follows: Epithelial (CDKN2A, EPCAM, CD24, CDH1), Endothelial (PECAM1, CDH5, ENG), Fibroblast (COL1A2, COL3A1, DCN), Myofibroblast (Simultaneous expression of fibroblast and smooth muscle cell markers), Smooth muscle cell (ACTA2, ACTG2, TAGLN), Lymphocyte (CD2, CD3D, CD3E, NKG7), Myeloid (CD163, CD68, LYZ, CSF3R), Mast (MS4A2, CPA3, TPSAB1), and Plasma (IGHG1, JCHAIN).
