# Supplementary material for: Glyoxalase 1: Emerging biomarker and therapeutic target in cervical cancer progression
Source: PLoS One. 2024 Jun 13;19(6):e0299345. doi: 10.1371/journal.pone.0299345 (PMC11175447; doi:10.1371/journal.pone.0299345)
Supplement: S2 Fig — (A) Volcano plot of average expression differences of proteins specifically expressed in cervical cancer and Lollipop plot of protein rankings from 0–100 in importance in a cancer prediction model. (B) Pan-cancer protein panel analysis of 7 selected proteins for cervical cancer prediction. (DOCX) [file pone.0299345.s002.docx]

**Supporting Information for**

**Glyoxalase 1: Emerging Biomarker and Therapeutic Target in Cervical Cancer Progression**

Ji-Young Kim^1^, Ji-Hye Jung^1^, Soryung Jung^2^, Sanghyuk Lee^2^, Hyang Ah Lee^3^, Yung-Taek Ouh^3,4^*, Seok-Ho Hong^1,5^*

^1^ *Department of Internal Medicine, School of Medicine, Kangwon National University, Chuncheon, Republic of Korea*

^2^ *Department of Life Science, Ewha Womans University, Seoul, 03760, Republic of Korea*

^3^ *Department of Obstetrics and Gynecology, School of Medicine, Kangwon National University, Republic of Korea*

^4^ *Department of Obstetrics and Gynecology, Ansan Hospital, Korea University College of Medicine, Gyeonggi, Republic of Korea*

^5^ *KW-Bio Co., Ltd, Chuncheon, Republic of Korea*

**
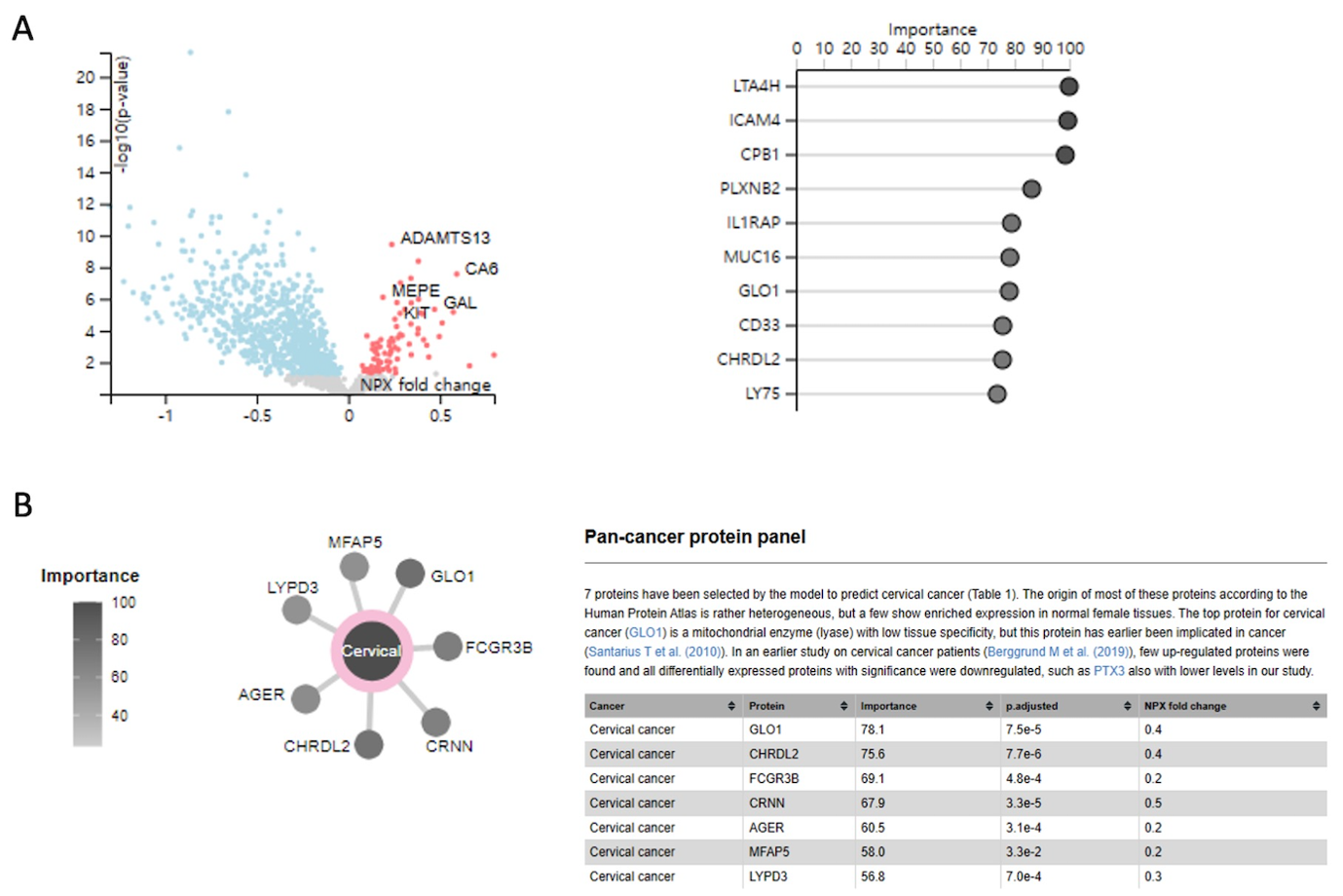
**

**Fig S2. Expression analysis of GLO1 protein in a predictive model of human cervical cancer.** (A) Volcano plot of average expression differences of proteins specifically expressed in cervical cancer and Lollipop plot of protein rankings from 0-100 in importance in a cancer prediction model. (B) Pan-cancer protein panel analysis of 7 selected proteins for cervical cancer prediction.
